# Heavy metals bioconcentration in *Crassostrea rhizophorae*: A site-to-site transplant experiment at the Potengi estuary, Rio Grande do Norte, Brazil

**DOI:** 10.1038/s41598-019-57152-w

**Published:** 2020-01-14

**Authors:** T. M. Senez-Mello, M. A. C. Crapez, C. A. Ramos e Silva, E. T. Silva, E. M. Fonseca

**Affiliations:** 1 Postgraduate Program in Dynamics of Oceans and Earth, Federal Fluminense University, Niteroi, RJ Brazil; 2 Center for Study of Water, Biomass and Oil (NAB), Federal Fluminense University, Niteroi, RJ Brazil; 3 Department of Oceanography and Limnology, Federal University of Rio Grande do Norte, Natal, RN Brazil

**Keywords:** Environmental impact, Biogeochemistry, Ocean sciences, Metals

## Abstract

In this study, we analyzed the bioconcentration of Cd, Cr, Cu, Pb, Ni, and Zn in the soft tissue of transplanted oysters in two sites in the Potengi estuary for six months. Native oysters collected before and after the transplantation experiment provided the background for statistical analyses. Cd, Cr, and Ni showed a strong inverse correlation with oyster weight in both sites. Transplantation upstream of the estuary presented increasing concentrations of Zn, Cu, and Pb and condition index (CI) and decreasing trends for Cd and Ni, whereas Cr oscillated significantly. In the downstream transplantation, Cu, Pb, and Zn and the CI tended to decrease, whereas for Ni, Cd, and Cr, the concentrations increased. Spatiotemporal principal component analysis correlated these results mainly with proximity to the polluting source, seasonality, and previous exposure to heavy metals. These results helped interpret the responses provided by these biomonitors to environmental changes, whether they are natural or anthropogenic.

## Introduction

Industrialization and urbanization are primary sources of heavy-metal contamination in estuaries and coastal ecosystems of tropical and subtropical countries (e.g., Brazil)^1,2^. Deforestation of estuarine margins also impairs the ability of the environment to withstand heavy-metal pollution, as mangrove forests are an important agent in the process of cycling organic matter and nutrients, acting as a “filter” by retaining and detoxifying harmful elements and substances^3–5^. It is a fact that metal pollution in aquatic systems will significantly increase in the future owing to the popularity of new technologies, such as nanotechnology, and the improper disposal of the so-called e-waste^6^.

Metals widely available in estuarine environments tend to be trapped in sediments and incorporated into the local food chain. Thus, the presence of heavy metals in sediments can induce toxic effects in living organisms when they exceed certain concentration limits^5^. These limits, according to Farrington *et al*.^2^, are dependent on the half-life of the metal itself (or the metallic compound) and its nature (whether essential or nonessential) and on the tolerance of the organism to the environment it is exposed to. Once discarded in an estuarine system, heavy metals can undergo various processes, such as dissolution, precipitation, adsorption, and complexation (with organic and inorganic dissolved ligands and particulate matter), and become deposited in bottom sediments^7^. These processes can create a potential source of pollution and adversely affect the environmental quality^8,9^.

Certain marine organisms, such as barnacles and bivalve mollusks, can metabolically accumulate large amounts of these metals in their tissues, having efficient strategies to tolerate toxicity and reduce damage in such a way that they can be employed in multipurpose environmental monitoring and several levels of environmental degradation, thus signifying the extent of pollution present in the adjacent water column^10,11^.

Bioaccumulation of heavy metals in oysters takes place through two fundamental mechanisms. The first mechanism arises from the formation of complexes between the heavy-metal ions and the functional groups of some enzymes that can block important metabolic processes performed by them. The second mechanism involves altering the structure of bivalve cell membranes when combined with some heavy metal. This combination may interfere with the transport of ions, such as Na^+^, K^+^, and Ca^−^, and substances essential for maintaining vital processes^12^.

The Potengi estuary region is extremely impacted by pollution^13–15^ and serves here as a study site to evaluate the bioconcentration of heavy metals (Cd, Cr, Cu, Pb, Ni, and Zn) in *Crassostrea rhizophorae* oysters. In this study, heavy metals were accessed through the total body load of approximately 300 soft tissue samples of oysters collected after transplantation between two estuarine sites, which already showed significant physicochemical differences^16–20^.

Generally, biomonitoring studies are performed using organisms collected at their original location. This methodology is indicated to detect pollutant levels from the surrounding environment^2,21^. However, the aim of this study is to simulate a major environmental modification (through exchange between sites) to understand how biomonitors react to it. Thus, the results obtained contribute both to the understanding of the mechanisms involved in the kinetics of heavy-metal uptake by *C*. *rhizophorae* oysters and to the creation of a knowledge base, enabling the use of this method in places where the sampling of native oysters is not possible, or in situations of true environmental changes.

## Materials and Methods

### Sampling site

**Figure 1:**
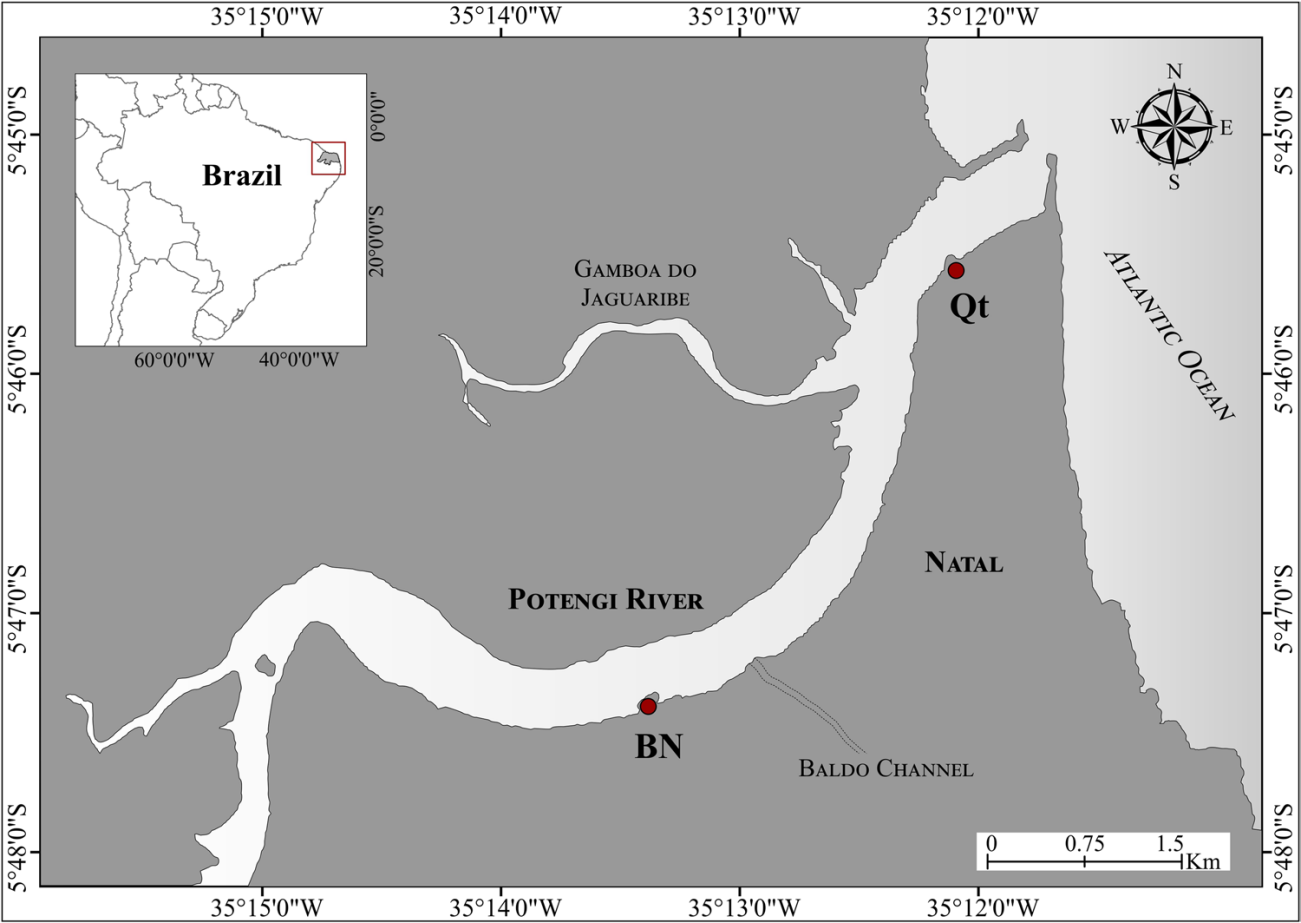
Site locations at Potengi. BN, Naval Base (Brazilian Navy pier); Qt, Quartel (Y-Beach, 17th Army Artillery Group). Map created by Senez-Mello, T.M., based on image captured with Google Earth Pro 7.3.2 software (Image catalog ID: 1010010004319001). Vectorized and edited by the author using CorelDraw (X8) software.

The Potengi estuary (Fig. 1), is inserted in the context of anthropogenically impacted areas, where heavy metals appear as one of the most important pollutants. Located in the northeast of Brazil, the main river, also called Potengi, runs approximately 180 km through industrialized areas, such as Natal, where its estuary is located^22^. Natal has about 774,205^23^ inhabitants, a very active port area, an oil terminal, and approximately 1,500 industries^24^. Almost 60% of domestic sewage is discharged untreated into the Potengi River and other smaller estuary contributors. However, Silva *et al*.^25^ pointed out that the estuary was already contaminated by the end of 1997, showing high concentrations of Mn, Fe, Cu, Ni, Pb, Zn, Cd, and Cr in the soft tissue of *Crassostrea* sp.

The first site, near the mouth of the estuary, was named Quartel (Qt; 5°45′30.6864″S, 35°12′5.475″W). There have been many studies reporting that the main introduction of pollutants in this site is done through Gamboa do Jaguaribe (Fig. 1), a channel levee with hydrodynamics dependent on the tide of the Potengi estuary. There, the original mangrove vegetation was removed for the installation of several shrimp farm fields and the riverside community. The pollutants that have been reported to be of major concern are Cu, Zn, and Cr compounds from the shrimp food diet and Cu compounds contained in fungicides and algicides used in the treatment of cultivation tanks^26^.

The other site, located upstream, was named Base Naval (BN; 5°47′20.4792″S, 35°13′21.2556″W). The introduction of pollutants in this place is due to the discharge of raw sewage made through Baldo’s channel (Fig. 1), resulting from immunization, domestic sewage, and the Industrial District of Natal (DIN). Both sites have heavy vessel traffic; however, the shipyard terminal is located at BN^13,25^.

### Experimental design

The experiment consisted of analyzing the biometrics and the heavy-metal content in the soft tissue of *C*. *rhizophorae* oysters reciprocally transplanted between two potentially contaminated environments located at the right margin of the Potengi estuary^25,27–29^ (Fig. 1).

The two sites are approximately 5 km apart from each other and were chosen on the basis of a previous experiment that yielded relevant results when comparing the morphology and metal concentrations (Cd, Cr, Cu, Ni, Pb, and Zn) among their native oysters ($N=20$)^30^. These results were then used to create a background scenario for the transplantation experiment. These initial native oysters are referred to herein as Nt0 (native oysters collected before transplantation). In addition, another native group ($N=30$) were collected by the end of the experiment and are referred to as NtF (native oysters collected after the end of the experiment).

The biomonitor species was chosen according to the criteria recommended by Rainbow^31^, such as the endemic presence of the organism and its relevance as an economic and food source for the local population.

The experimental plan was designed to answer the following hypotheses:

- Significant differences in metal concentrations and biometrics between native oysters and transplanted oysters.
- Significant differences in metal concentrations and biometrics achieved by transplanted oysters versus native ones collected at the same site by the end of the experiment.
- Principal factors influencing such variations. The following variables were considered: sampling site, oyster conditions before transplantation, impact of the transplant itself, and seasonality in synergy with the physicochemical characteristics of each site.

### Field methodology

The transplantation process consisted of transferring 150 native oysters from Qt to BN and vice versa. For this, the already existent epifauna was carefully removed and oysters were housed in bag-like cages made of polyvinyl mesh, containing about 30 individual each. Every 15 days, 15 oysters were picked from the cages at each site and taken for biometric and metal analyses. The experiment lasted six months, totaling 11 transplantation campaigns (T1–T11), as well as an additional sampling of native oysters before and after the experiment. Sampling was performed according to Roberts and Elliott^32^ through the criterion of size equivalence of individuals in the same population.

### Laboratorial analysis

Immediately after each collection, oysters were identified and preserved on ice for transport to the laboratory. Aliquots for metal analysis were prepared according to Silva^25^, who used the total soft tissue (meat) of the oyster after undergoing an oven dehydration process at approximately 80° C until a constant weight was obtained, referred to in this study as “dry weight” (d.w.). Determination of metal concentration was performed using inductively coupled plasma–optical emission spectrometry (ICP-OES)^33^ as described by the authors above. Additionally, the biometrics of each individual were recorded for subsequent calculation of the condition index (CI): CI = (soft tissue d.w./shell volume) × 100^34,35^. The results are expressed in units of g/cm³ and are directly proportional to oyster health.

Analytical quality was periodically tested using Standard Reference Material (SRM-NIST 2976 mollusk tissue), as well as procedural blanks to identify possible contamination. Table 1 presents the SRM certified values and the SRM measured values throughout this study. The recovery percentage of all analytes ranged from 80% to 110%. All analyses were conducted at the Center of Studies in Oil and Natural Gas, Federal University of Rio Grande do Norte, during the year 2007/2008.

**Table 1:** Analytical quality. Certified and measured values for the SRM NIST 2976 (mean ± s.e.m., µg.g^−1^ of d.w.).

Metal	Certified SRM	Measured SRM
Cd	0.82 ± 0.16	0.56 ± 0.08
Cr	0.50 ± 0.16	0.58 ± 0.30
Cu	4.02 ± 0.33	3.39 ± 1.00
Ni	0.93 ± 0.12	0.85 ± 0.22
Pb	1.19 ± 0.18	0.79 ± 0.19
Zn	117.0 ± 13.0	118.7 ± 18.46

Atmospheric and tidal data were provided by the Hydrographic Center of the Brazilian Navy through the Data Exchange Sector recorded at the buoy station “Trapiche CPRN” (05°46.7′S, 35°12.5′W) over the year 2007 (Supplementary Fig. S1).

### Statistical analysis

All statistical analyses were performed using Statistica software (v.13). The assumptions for the parametric analysis (normality and homoscedasticity) were met after the exclusion of the outliers (±3^∗^ standard deviation) and data normalization (log_10_). Metal concentrations from native oysters were compared using Student’s *t*-test^30^.

Temporal analyses for biometrics and heavy metals found for *C*. *rhizophorae* samples at the two sites were performed using analysis of variance (ANOVA). All results were reported at a 95% confidence level (CL) and were considered significant when $p < 0.05$.

The dependence of the metal concentrations on the oyster d.w. (size effect) was tested in order to correct the influence of this parameter^27,31^. This test was performed via double-log regression^31,36–38^:

${\log }_{10}\,y={\log }_{10}a+b{\log }_{10}x$, where, $y$ is the metal (µg. g^−1^), $x$ is the dry weight (g), $a$ is the intercept, and $b$ is the regression coefficient.

In cases in which the covariance showed significant values ($p < 0.05$), the statistical method used was the analysis of covariance (ANCOVA), which considered the $x$-values equal for all samples ($x=\,{\log }_{10}$ mean d.w. of the population), from which the metal concentrations were calculated. Tukey’s honestly significant difference (HSD) post hoc test (for samples of different sizes) was applied to identify the highest contrasts within campaigns.

Finally, spatiotemporal principal component analysis (st-PCA)^39^ was performed to illustrate the main correlations between the parameters and heavy metals. In this way, the following steps were followed: (a) previous analysis of data through descriptive statistics (Supplementary Table S2), (b) data health check to remove outliers and variable redundancy, (c) log-normalization of the dataset, (d) correlation matrix selection, (e) retention of factors with higher eigenvalues, and (f) considering heavy metals as active variables in case distributions while electing environmental ones as supplementary.

## Results and Discussion

### Water and sediments

The physicochemical parameters and concentrations of heavy metals from water columns and sediments, as well as granulometry, were compiled in Table 2. For more details, these data can be accessed through the Institutional Repository of the Federal University of Rio Grande do Norte^17,40^. Analysis of the water column demonstrated that, at Qt, the salinity, turbidity, pH, and dissolved oxygen (DO) were higher compared to the upstream site (BN). The temperature, biochemical oxygen demand (BOD), and total organic carbon (TOC) were higher on the uppermost site (BN) than downstream. These results are in line with the local estuarine dynamics in which tidal intrusion acts more intensely near the mouth of the estuary and gradually decreases upstream^41^. With the exception of the BOD, all other parameters cited above were within the standards for water quality proposed by the Brazilian legislation for saltwater (salinity > 30‰), where recreational and fishing activities take place^42^. In both sites, the ammoniacal nitrogen (NH_3_) was above the limit proposed by CONAMA^42^ resolution, whereas nitrates had a high concentration in BN. No nitrites were detected.

**Table 2:** Sites characterization and reference values. Water column, sediments, and native oysters from the sampling sites (Qt and BN). Heavy metals and biometrics from the oysters are presented as the mean ± 1 standard deviation from the mean. ^*^Significantly different according to Student’s *t*-test when ${\boldsymbol{p}} < 0.05$.

Medium	Unit	Variable	Qt	BN	Reference Values	
Water^40^	‰	Salinity	32.5	31	>30	CONAMA^42^
	NTU	Turbidity	5.5	4.0	—	
	°C	Temperature	26.5	27.05	—	
	Total	pH	7.7	7.6	6.5–8.5	
	mg. L^−1^	DO	8.3	7.1	>5	
		BOD	3.25	3.75	≤5.0	
		TOC	20.50	41.50	<5.0	
		NH_3_	0.64	0.42	<0.4	
		NO_3_^−^	0.19	0.31	<0.4	
		NO_2_^−^	ND	ND	<0.07	
		N_total_	0.495	0.765	—	
		Cd	0.006	0.006	<0.005	
		Cr	ND	ND	<0.05	
		Cu	ND	0.03	<0.005	
		Ni	0.05	0.05	<0.025	
		Pb	0.015	0.03	<0.01	
		Zn	0.035	0.155	<0.09	
Sediment^17^	m	Depth	4.70	7.8	—	TEL CCME^44^
	%	Granulometry^72^	Silty sand	Sandy silt	—	
	mg.kg^−1^	Cd	0.050	0.083	0.7	
		Cr	51.67	111.0	52.3	
		Cu	12.13	47.27	18.7	
		Ni	17.05	41.15	15.9	
		Pb	14.67	32.21	30.2	
		Zn	55.0	101.0	124	
*C*. *rhizophorae*	g	d.w.*	0.12 ± 0.04	0.24 ± 0.07	—	Min–max (oysters from Potengi)^25,29^
	cm	Shell	3.13 ± 0.32	4.0 ± 0.79	—	
	(g/cm³)	CI*	3.79 ± 1.19	6.11 ± 1.47	—	
	µg. g^−1^ d.w.	Cd*	0.71 ± 0.15	0.18 ± 0.03	1.34–2.12	
		Cr*	0.54 ± 0.26	0.67 ± 0.20	0.9–3.9	
		Cu	79.03 ± 11.05	88.92 ± 29.32	25.2–161	
		Ni*	1.85 ± 0.50	1.12 ± 0.20	1.2–2.32	
		Pb*	0.27 ± 0.05	0.34 ± 0.07	2.3–7.1	
		Zn*	1,296 ± 244.7	1,995 ± 357.1	967–3,104	

Regarding metals in water, Cd and Ni presented similar values for both sites, which were above the reference limit. Cr presented values below the detection level. Cu, Pb, and Zn were detected in larger quantities in BN and were all above the water quality reference limit (Table 2).

Evaluations based on heavy metals in water have some limitations. In addition to tiny concentrations, often close to detection limits, the amounts can vary rapidly with seasonal changes, time of day, and freshwater runoff ^27^. Acidity and organic matter are known to be important factors in determining the fate of heavy metals in aquatic systems.

Sediment analysis classified Qt as having silty-sand granulometry, whereas at BN (with a higher percentage of fines), granulometry was classified as sandy-silt^43^ (Table 2). BN was the site that presented higher concentrations for all metals; with the exception of Cd and Zn, the values were above the threshold effect level (TEL) for marine sediments, as proposed by Canadian Council of the Environment (CCME)^44^, indicating the possible emergence of ecotoxicological effects in the biota location. Metals can be transferred from sediment to water due to an increase of the salinity, changes in sediment redox status, pH decrease, and the presence of organic complexing compounds. The first three processes release “free-metal” in the form of mixed complexes with small inorganic ions and water^45^.

### Native oysters

Comparing native oysters from Qt and BN, significant differences were revealed between these two sites. The following results refer to mean values. The concentrations of heavy metals are shown in μg/g of oysters’ soft tissue d.w. Among the biometric data, the d.w. and the CI were higher for BN oysters (d.w. = 0.24, CI = 6.11) than for Qt oysters (d.w. = 0.12, CI = 3.79). However, the lengths of the shells exhibited no significant difference (Qt = 3.13, BN = 4.0) (see Table 2 for the standard deviations). These results show that the sampling methodology that was performed according to the equivalence criterion of the average population size (see Section 2) was efficient in minimizing the possible unwanted influences of other parameters related to the development and reproductive stage of mollusk in metal accumulation. It is interesting to note that even when “standardizing” samples through the size of the shells, it was possible to find significant differences for d.w. and CI, endorsing the importance of this index as an integrated measure of the general health of oysters, as already demonstrated by several authors^34^. Among the metals, the highest means of Cd and Ni were found for Qt native oysters (Cd = 0.71, Ni = 1.85) when compared to BN (Cd = 0.18, Ni = 1.12). For Cr, Cu, and Zn, the highest means were obtained for BN native oysters (Cr = 0.67, Cu = 88.92, and Zn = 1,995) when compared to the native oysters of Qt (Cr = 0.54, Cu = 79.03, and Zn = 1,296). However, Cu was twice above the upper limit for human consumption.

### Transplanted oysters

#### Size effect

**Figure 2:**
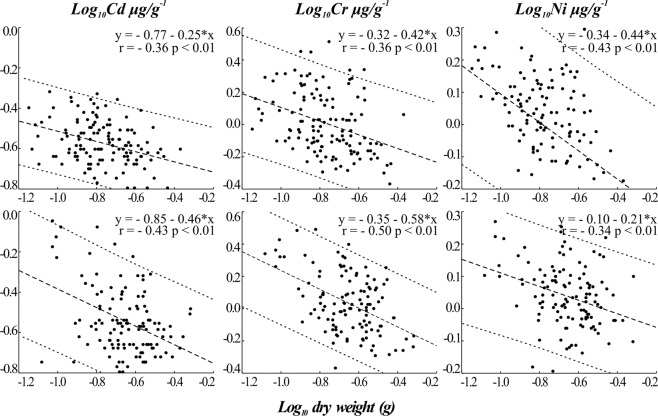
Size effect results. Linear regression between log_10_ d.w. (g) and log_10_ metal concentration (µg/g). Upper graphs: Cd, Cr, and Ni from transplanted oysters at Qt. Lower graphs: Cd, Cr, and Ni from transplanted oysters at BN.

According to the regressions made between metals and soft tissue d.w. (Fig. 2), both sites showed similar results, presenting size effects for Cd, Cr, and Ni but not for Cu, Pb, and Zn. Cases in which the covariance hypothesis was accepted are presented below, justifying the use of ANCOVA.

For Cd and Cr, a stronger correlation with d.w. can be observed at the uppermost site (BN), whereas for Ni, this occurred at the lower site (Qt). All correlations were inversely proportional; thus, the smaller the oyster, the higher the metal concentration. Some authors have already reported the size effects for these metals^46–48^. Rainbow and Moore^43^ showed recurrent cases in which smaller organisms presented higher metal concentrations when compared to larger individuals of the same species. These authors attributed this effect to the fact that smaller organisms have a greater absorption area per gram of body weight relative to the bigger ones.

Another factor pointed out by Rainbow^27^ as a probable explanation is the age of the individual, as younger ones have more active metabolism and, thus, higher filtration rates. Yet, the literature points to a dilution effect in which concentrations seem to be lower as the oysters grow as being relevant. For this, we should assume that the bioaccumulation rates cease or decrease over time, which was not the case here as we have shown.

More studies on the size effect are needed in this field, and we recommend testing whenever possible in a way to elucidate this covariation. For now, it is not clear why in certain cases the size effect is not observed; therefore, it is recommended to verify whether or not this effect matters, making the interpretation of data more reliable^25^.

#### Condition Index

**Figure 3:**
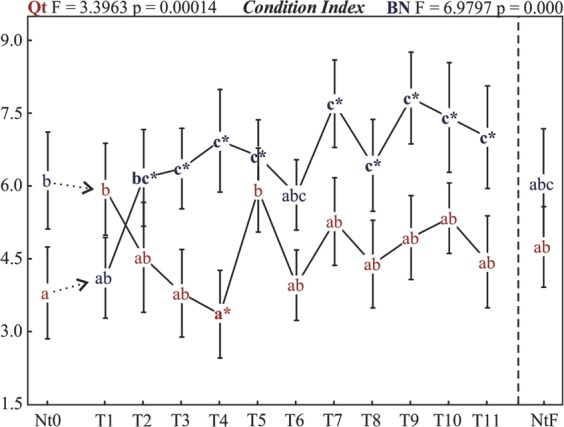
Condition Index. Letters are placed at the means, and the vertical bars denote 95% CL. Significant differences are estimated by a priori ANOVA. Samples sharing the same letters are not significantly different. The symbol *shows samples with significant differences from Nt0 (native oysters before transplantation) according to Tukey’s HSD post hoc test.

Upward transplantation (Qt to BN) showed significant differences from native oysters right before 45 days of relocation (T2, blue; Fig. 3). Thereafter, it continued in a positive trend, denoting stability in the development of the oysters. There were only two exceptions in this trend: the 1st and the 6th campaigns (T1 and T6), both presenting values similar to those of the initial and final native oysters (Nt0 and NtF). The highest CI was reported at T9 (Fig. 3, blue).

Transplantation made in the downstream (Qt) direction presented CI values successively lower in comparison to Nt0, and values became significantly different at T4, reaching the minimum for this experiment. Thereafter, CI tends to increase again until it reaches values similar to those of native oysters. From T4 onward, a trend can be noticed for both sites, where variations occur in the same direction.

Comparing these two groups, there was an opposite tendency well stated by the intersection of the two lines (T1-to-T2 interval, Fig. 3). At the end of the analysis, oysters at Qt showed a worsening CI during the initial 75 days (T4) followed by an improvement. Despite the subsequent oscillations, at the end of the experiment, oysters transplanted to BN had a higher CI compared to the initial and final native oysters.

Typically, oysters accumulate glycogen before winter and use these reserves for gametogenesis in the next season^49^. A sudden drop in CI caused by spawning was reported in a study in Todos os Santos Bay (NE, Brazil), where authors pointed out that in tropical environments, spawning is not a seasonal event^50^. However, it can still be considered a key factor that causes differences in oyster conditions throughout the year. For the oyster *Crassostrea rhizophorae*, gametogenesis is a continuous process, homogeneous within the population, with spawning occurring in partial discharges during the entire year, with peaks every 3 months^51^ explaining the smaller variation and similar trends found in Potengi. However, between T1 and T6, IC varied considerably within sites, demonstrating the likely predominance of other factors rather then spawning.

#### Heavy metals

**Figure 4:**
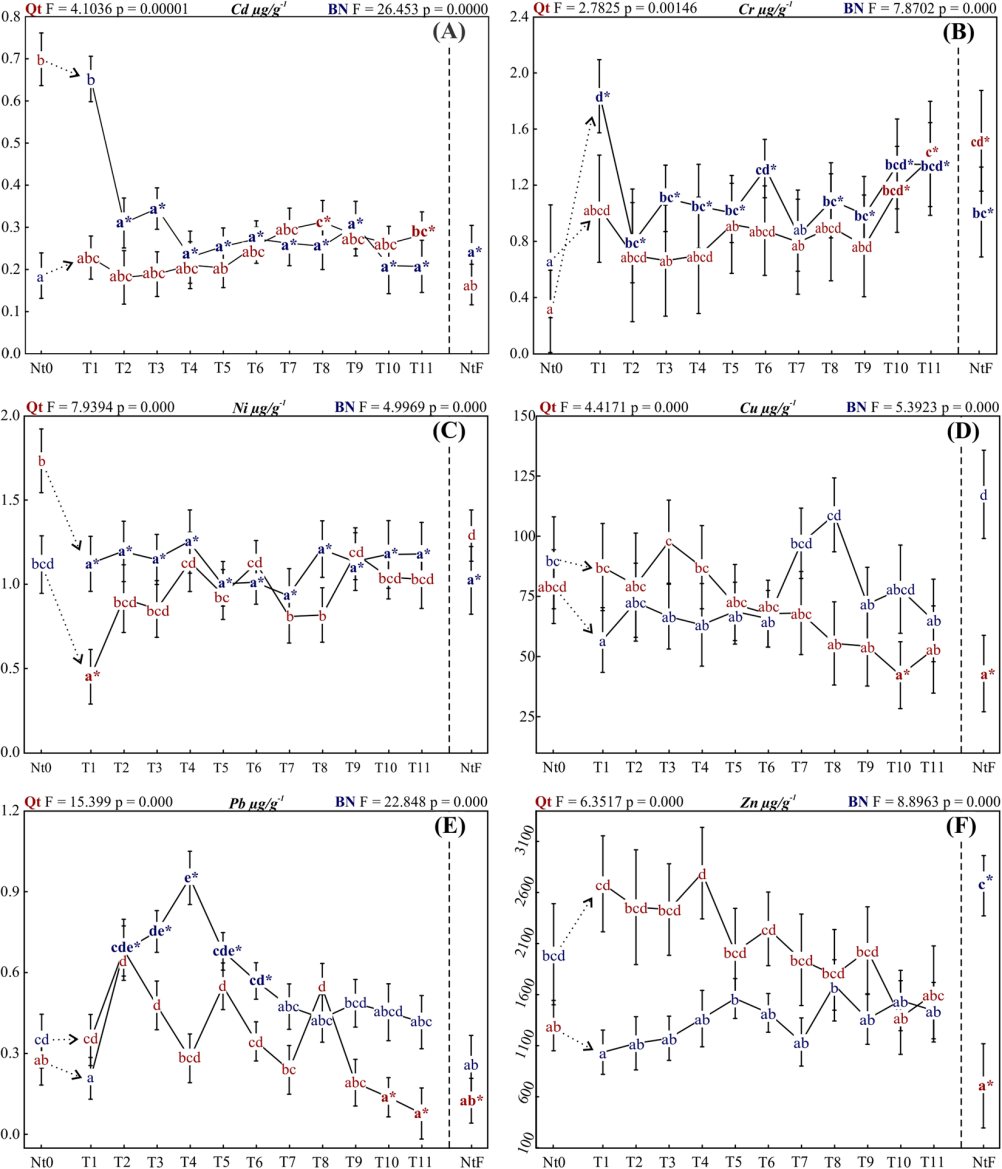
ANCOVA and ANOVA. Letters are placed at the means, and the vertical bars denote 95% CL. Significant difference were estimated by a priori ANCOVA for (**A**) Cd, (**B**) Cr, and (**C**) Ni at covariate means (Qt: log_10_ d.w. = −0.7597, BN: log_10_ d.w. = −0.6791). For the other metals, that is, (**D**) Cu, (**E**) Pb, and (**F**) Zn, differences were estimated by ANOVA. Samples sharing the same letters are not significantly different, and the asterisks show the ones with a significant difference when compared to Nt0 (native oysters before transplantation) by Tukey’s HSD post hoc test.

Cd: Native oysters from Qt, when transplanted to BN presented a rapid and sharp decline with significantly lower values at T2 (Fig. 4A, blue). After that, the differences were not significant, but the concentrations continued to decrease until the end of the experiment (T11), at which point the lowest value throughout the entire experiment was found. While transplantation from BN to Qt, the results were more homogeneous. There was a maximum peak concentration at T8 followed by decrease and then another smaller but significant peak (T11), at which the concentrations were above those found for Nt0 (Fig. 4A, red).

Although both sites had concentrations above the recommended limit for water, the sediment was within the proposed quality standard (3.1), showing that the bioavailability of Cd observed through the Qt’s Nt0 had an influence of factors other than just the proximity with the source, since among the transplanted oysters none reached similar concentration.

Cr: One month after transplanting oysters to BN, the Cr values increased significantly, reaching the highest concentration throughout the experiment (Fig. 4B, blue). However, after another 15 days (T2), this group revealed reduced values and remained significantly higher until the end of the experiment (the only exception was T7). At the end, the final value (T11) was equivalent to that found for NtF from BN and Qt.

Oysters transferred to Qt showed no significant difference until T10 sampling. After that, there was a significant increase, when the last sample (T11) presented similar values to NtF from Qt and BN and a significantly higher value compared to Nt0 (Fig. 4B, red). Both transplantations increased the concentration of Cr after T9, which coincided with the dry season period.

Cr concentrations in the water of both sites were below the detection limit while in sediments, the values were considered high, where BN presented approximately twice the concentration from Qt. Reflecting the concentrations found in sediment, oysters transplanted for BN showed increased concentrations, but still within the range already reported for oysters in this estuary^25,29^.

Ni: All oysters transplanted to BN showed significantly lower concentrations compared to Nt0 (Fig. 4C, blue). One month after transplantation (T2), Ni was reduced by almost a half, followed by nonsignificant variations until the end of the experiment.

At Qt transplantation, Ni initially (T1) decreased to a lower value compared to Nt0; however, from the following collection (T2), the values increased again, showing no more significant differences when compared to native oysters (Fig. 4C, red). At the end of the experiment, the Ni values were equivalent to those of both native oysters (Nt0 and NtF).

As shown (Table 2), both sediment and water concentrations were above the values that regulate environmental quality while, Ni from transplanted oysters remained consistent with those already reported for the Potengi. Bioconcentrations were below expectations if compared with the levels in the environment, however, it is known that oysters have a greater affinity for zinc, copper and silver than other metals^52^. When comparing these two transplants through graphics, it was noted that oysters presented a similar accumulation behavior between Ni and Cd.

Cu: Oysters transplanted to BN showed no significant differences when compared to Nt0. However, T7 collection presented significantly higher values compared to T1, and T8 was significantly higher than the rest of the experiment, except for T2 and T10 (Fig. 4D, blue). Cu found in NtF oysters was similar to Nt0, T7, T8, and T10 and higher than the rest. Compared to T11, NtF had almost twice the concentration.

At the downstream site (Qt), Cu depurated throughout the transplant and reached at T10 values significantly lower than those at NT0 and compatible with NtF (Fig. 4D, red). Overviewing the graph (Fig. 4D), it is possible to see an opposite tendency between the two transplants.

In Qt Cu was not foi detected in water and presented value below the threshold effect limit in the sediment. In BN, both compartments presented values above the recommended. Transplanted oysters reflected well the levels of contamination of the environment, bioaccumulating Cu in BN and lowering bioconcentrations of this metal in Qt. Since this metal is considered essential, metabolic variations need to be considered in conjunction with environmental ones^45^.

Pb: Both experiments showed increasing values until T2, but then the oysters transferred to Qt started to depurate, whereas at BN the values continued increasing (Fig. 4E). At T4, the oysters at BN reached the maximum Pb concentration for the whole experiment; from this sampling onward, Pb depurated, reaching a value similar to that found at Nt0 and NtF (Fig. 4E, blue).

Oysters transplanted to Qt (Fig. 4E, red) showed an oscillating and nonsignificant variation, with concentrations increasing and decreasing over similar time intervals. From T9 onward, Pb showed values significantly lower than those of Nt0 and similar to those of NtF.

On both sites the lead content in water were above to the recommended concentration, and only in BN the sediment was considered contaminated. Comparing to the previous studies oysters had considerably lower values, showing an improvement in environmental quality with regard to this metal.

The process of absorption of Pb from environmental sources depends on its bioavailability as well as physical and chemical status, in addition to factors related to the intoxicated organism, such as the age, physiological status, nutritional condition, and genetic factors^5^.

Pb presents affinity for sulfhydryl groups (HS), amino radicals (NH_2_), radical hydroxide (OH), and also phosphoric acid (H_3_PO_3_), forming complexes with endogenous compounds that interfere with cellular functions. The latter is a commonly used reagent in the soft drink industry present around Potengi, where cases of untreated tailings have already been reported by the media^53–55^.

Zn: Oysters transplanted to BN did not present values different from those of Nt0. However, when the samples were compared to T1, the increase was significant, with T5, T8, and T10 (Fig. 4F, blue) showing increased values.

After transplanting oysters to Qt, Zn showed a tendency to decline throughout the experiment; however, the variances around the averages do not allow affirming that these differences within samples are statistically significant, whereas sample T10 was significantly smaller than T1, T4, and T6 (Fig. 4F, red) and equivalent to NtF.

In both transplants, Zn showed no significant differences when compared to Nt0; however, the differences were substantial when compared to the final native samples (NtF). In addition, the latest samples from both experiments (T10 and T11) had very similar values (Fig. 4F).

### st-PCA

The metals deposited in the aquatic environment can come from the atmosphere or from soil leaching or direct dumping of contaminants. As a general trend, metal is rapidly divided between the sediment and the aqueous phase, depending on the pH of the water, the salts dissolved in it, and the presence of organic complexing agents^56^. When there is a reduction in pH, the natural process of leaching and the availability of most metals can be intensified^6^.

In this way, the importance of environmental elements, such as rain, wind, tide, evaporation, insolation, and atmospheric temperature, was evidenced by st-PCA, which retained only those of greatest influence to aid in the interpretation of variances found by ANOVA/ANCOVA. Next, biplot graphs showed the temporal transition between the characteristics found between initial native oysters, transplanted oysters, and final native oysters.

**Figure 5:**
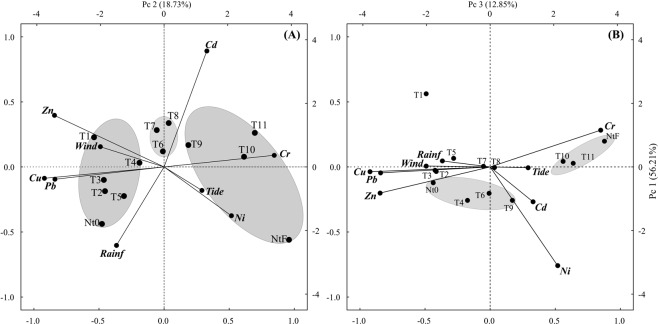
Qt st-PCA. (**A**) Principal components 1 × 2 and (**B**) principal components 1 × 3 of the transplant experiments. Metals represent the average concentrations found in oysters for each sampling time. Wind, precipitation, and tide were calculated from the average of two weeks prior to each sampling campaign.

The first three st-PCA components for Qt transplantation (Fig. 5A,B) cumulatively explained 87.8% of the variance. The first component (Fig. 5A, 56.21%), showed three groups of oysters with distinct characteristics. The first group on the negative side of this axis was composed of the first five samples (T1, T2, T3, T4, and T5) and the Nt0. The most important metals presented for these oysters were Cu, Pb, and Zn (in order of importance), whereas the main environmental variables influencing metal uptake were the wind and the rain. This axis describes the initial state of native oysters from BN, where there is intense port activity and direct impact from rain and wind, mainly because the samples were collected during the winter, when the highest precipitation rates were recorded. The opposite side of this axis represents the oysters in the final stage of the experiment (T9, T10, T11, and NtF). The main metals contained in these oysters are Cr and Ni, related to which are the drought period and consequently the greater influence of tides entering the estuary. Yet, it is possible to observe a third group (T6, T7, and T8) plotted in the intermediate portion of the graph composed of transient characteristics between the initial and final samples. The ANOVA that was presented here earlier indicated that the oysters transplanted to Qt exhibited an initial drop in the CI and that the most impacted group was precisely this intermediate group, which possibly exhibited a great energy loss to adapt to the new physicochemical conditions of this highly dynamic environment.

In the second component (18.73%, Fig. 5A), Cd appears to be the most relevant factor underlining the ecotoxicological effect through the weight loss of the intermediate samples, in which the oysters begin to depurate Zn (an essential metal) while accumulating Cd, hence exceeding the value found for the native oysters there (NtF) at the end of the experiment. It is likely that the previous exposure to Zn induced the largest accumulation of Cd, as the main detoxification mechanism for both metals is the same, that is, induced metallothionein production by increased plasma concentrations of these pollutants^57^.

The third component (12.85%, Fig. 5B) shows that the first sample after transplantation had the lowest Ni concentration throughout the entire experiment, but then this metal began to accumulate again until the last two samples (T10 and T11), assuming values similar to those of NtF. The same is true for transplantation in the opposite direction, with the lowest concentration of Ni at T1. The main factor influencing this behavior is unclear and can be attributed to an external condition as it was similar for both transplants. Further studies are needed to understand this initial clearance at both sites.

**Figure 6:**
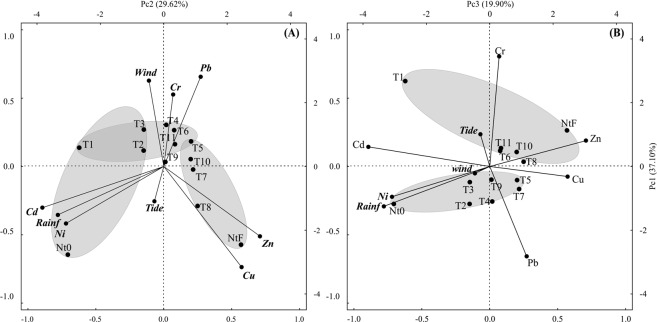
BN st-PCA. (**A**) Principal components 1 × 2 and (**B**) principal components 1 × 3 of the transplant experiments. Metals represent the average concentrations found in oysters for each sampling time. Wind, precipitation, and tide were calculated from the average of two weeks prior to each sampling campaign.

For the other transplant experiment, PCA (Fig. 6A,B) explained 86.6% of the overall variances. The first component (37.10%, Fig. 6A) showed higher values of Cd and Ni for native oysters from Qt and a tendency to depurate this metal once they are transplanted to BN. The only samples with a significant correlation with rainfall were Nt0 and the first three ones (T1, T2, and T3), because they were collected during the winter; as they are native oysters, the values of Cd and Ni were probably reached during the last dry season. Positively loaded on the first component (Fig. 6A), Zn and Cu are the most important variables presenting increasing values over time (T5, T7, T8, T10, and NtF) and characterizing the changes suffered by the transplanted oysters, which also detoxified Cd and Ni. Similar to the downstream transplant, there is a transition group in the middle part of this axis. In this case, PCA demonstrated the importance of proximity to the source of pollution, overriding the influence of seasonality. ANOVA was fundamental to understand that the significance of the rain factor, in this case, was due to the sampling rate. Actually, variance analysis showed that Cd is negatively correlated with rain and tends to increase during the dry season^47^, perhaps as an effect of the saline wedge advancing upstream in the BN direction.

The second component (Pc2 29.62%, Fig. 6A) showed a positive correlation between the intensity of wind and the Pb and Cr content in oysters. As wind is an important feature of the Brazilian northeastern coast throughout the year, a more careful analysis taking into consideration the concentrations present in the water column should be made. However, it is not difficult to assume that wind is partly responsible for introducing air pollutants from industrial districts into the water column. Negatively loaded on the second component, Cu and Zn are present in the final native oysters in higher quantities (almost twice) compared to the transplanted ones. The preexposure of Qt oysters to Cd seems to be a determining factor in Cu and Zn accumulation after being transplanted to BN. A similar behavior was observed by Liu and Wang^57^. The evidence for this is that, in these oysters, although the concentrations of these two metals increase over time, the values presented at the end of the experiment represent approximately half of those found in the final native oysters. This may be because the half-life of Cd is known to be longer^45^ than that of Zn and Cu and because these metals compete for the same binding site in detoxifying proteins such as metallothionines and glutathiones^58,59^. Transplanted oysters require longer exposure times to exhibit concentrations similar to those of oysters not exposed to this contaminant.

Finally, the third axis (19.9%, Fig. 6B) presents a more detailed view of these two metals, in which, despite showing increases relative to native Qt samples, Pb increased in freshly transplanted samples, whereas the increase of Cr was more intense at the T6 sampling. This axis also shows that the sudden increase in Pb is related to the rainy season, which, through runoff, introduces this pollutant in greater quantities into the estuary.

Importantly, as the salinity within a tidal estuary can be quite variable, the data used in the PCA refer to a biweekly average of tidal range and accumulated precipitation. These two factors, in addition to directly influencing the introduction of pollutants and estuarine currents, when interpreted together, may indicate a shift of the saline wedge in both directions within Potengi. The results shown here are consistent with previous studies^18,22,60^, showing that although tides can reach great distances within this estuary, the highest salinity is restricted to the first portion preceding BN.

The literature emphasizes the abiotic importance through local hydrodynamics, the chemical form of pollutants, the presence of other substrates competing for binding sites, and the physicochemical composition of the adjacent medium^27,61,62^, whereas the biotic ones include the life-cycle stage, gender, specific genetics, and physiological conditions^63–66^. In this study, we found that the variations appear predominantly as a combined effect of the biomonitors’ proximity to the source of pollution, seasonal and hydrodynamic variation, and prior exposure of the biomonitor to metal-contaminated environments. These results are consistent with other published studies^1,67–69^.

## Conclusions

By comparing the CIs of the upstream and downstream transplanted oysters, it can be deduced that transplantation alone does not have a detrimental impact on bivalves. This is primarily determined by the conditions found in the transplantation site.

The wide-ranging rate of variation in metal bioaccumulation shown in this study has been attributed to the synergy between biotic and abiotic factors^70^. Previous studies on molecular analysis of oysters^37,58,59,71^ showed how the metal content impacts their metabolism and, therefore, the CI. Here the oysters’ d.w. and CI served as a biotic indicator of their general health status due to metal accumulation.

The higher amounts of Zn, Cu, and Pb from native BN oysters, as well as from those transplanted at the end of the experiment, showed that these heavy metals are pollutants of great concern in this region and are directly related to the winter period owing to the urban runoff of stormwater to the aquatic environment.

For metals like Pb, Cr, Cd, and Ni, oysters appeared to respond faster to changes induced by transplantation; therefore, the last transplanted samples presented concentrations similar to those of the final native ones. The same was not true for Zn and Cu, and it is possible that, in such cases, longer-term exposure is needed until the oysters acquire similar values to those that have never been transplanted.

Yet, in BN, transplantation showed that although the oysters accumulated Zn and Cu throughout the experiment, the values reached by the final sampling (T11) were significantly lower than those from native oysters. Thus, based on the important influence of rain on the explanation of variances, it can be deduced that the exposure period was not sufficient for transplanted oysters to reach values similar to those of the native ones, as the latter had already accumulated these metals during the previous winter. As these two metals (considered to be essential) are directly linked to the oysters’ reproductive cycle, caution is advised when interpreting results showing reduced concentrations for Cu and Zn.

Here, different statistical approaches revealed that, despite their distinct analytical power, the results were complementary. While ANOVA presented detailed results for metal concentrations one by one, spatiotemporal PCA allowed a “macro” overview of the environmental variations acting over transplanted oysters.

## Supplementary information

Supporting Information.

Supporting Information 2.
