# Supplementary material for: Heavy metals bioconcentration in Crassostrea rhizophorae: A site-to-site transplant experiment at the Potengi estuary, Rio Grande do Norte, Brazil
Source: Sci Rep. 2020 Jan 14;10:246. doi: 10.1038/s41598-019-57152-w (PMC6959329; doi:10.1038/s41598-019-57152-w)
Supplement: Supplementary file 1 — Supporting Information. [file 41598_2019_57152_MOESM1_ESM.pdf]

Heavy metals bioconcentration in *Crassostrea rhizophorae*: A site-to-site transplant experiment at the Potengi estuary, Rio Grande do Norte, Brazil

Atmospheric and tidal data used to perform the spatiotemporal principal component analysis (st-PCA).

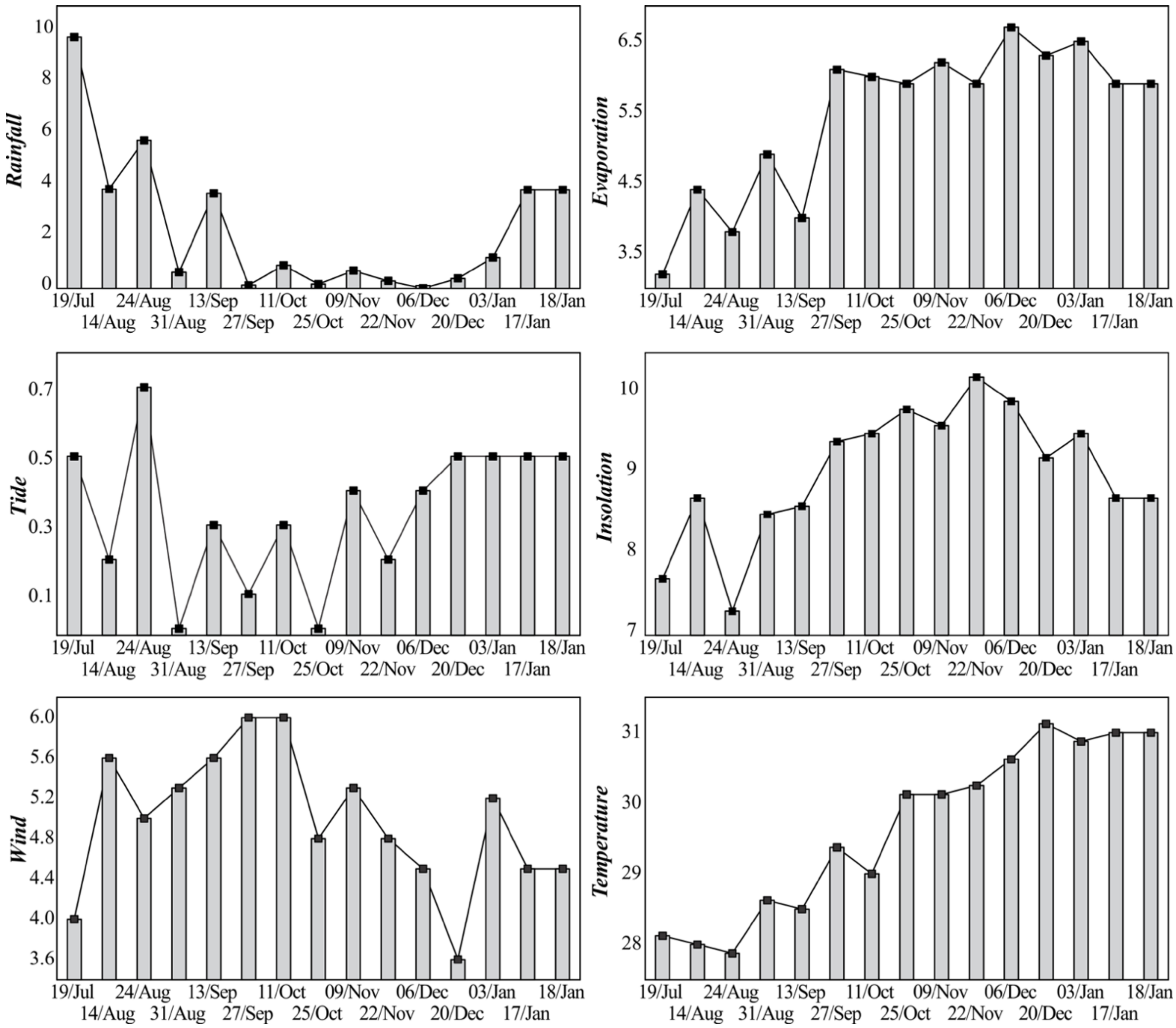

Figure S1: *Data provided by the Hydrographic Center of the Brazilian Navy through the Data Exchange Sector over the year 2007. Rainfall – mm/m^2; Evaporation – Piche's point mm/d; Tide – meters; Insolation – n (hour); Wind – meters/second.; Temperature – Celsius degree.*
